# Chemotherapy/Radiotherapy-Induced Dysphagia in Head and Neck Tumors: A Challenge for Otolaryngologists in Low- to Middle-Income Countries

**DOI:** 10.1007/s00455-024-10756-5

**Published:** 2024-09-24

**Authors:** Álvaro Gómez, María Alejandra García-Chabur, Daniel Peñaranda, Antonieta Gómez-Mendoza, Juan Carlos Forero

**Affiliations:** 1 Otolaryngology Department, Fundación Universitaria de Ciencias de la Salud - Hospital de San José, Bogotá, Colombia; 2 Surgery Department, Fundación Universitaria de Ciencias de la Salud - Hospital de San José, Bogotá, Colombia; 3 Otolaryngology Department, Fundación Santa Fe de Bogotá, Bogotá, Colombia

**Keywords:** Dysphagia, Chemotherapy, Radiotherapy, Cancer, Head and Neck Cancer

## Abstract

Head and neck cancer accounts for 2.8% of all cancers and a large proportion of these patients have a locally advanced stage of the disease, for which chemotherapy and radiation therapy are potentially curative treatments. Dysphagia is one of the most common chemoradiotherapy-related side effects in head and neck cancer since it can lead to life-threatening complications. Reports from the current literature suggest better swallowing outcomes with intensity-modulated radiotherapy (IMRT) compared to three-dimensional conformal radiotherapy (3DCT). However, in low-/middle-income countries, multiple healthcare access barriers to 3DCT that may lead to higher rates of chemo/radiotherapy related adverse events. This narrative review provides a comprehensive appraisal of published peer-reviewed data, as well as a description of the clinical practice in an otolaryngology referral center in Colombia, a low-income country.

## Introduction

Head and neck cancer accounts for 2.8% of all cancers and a large proportion of patients have a locally advanced stage of the disease [1]. Chemotherapy and radiation therapy are potentially curative treatments for locally advanced head and neck tumors [2]. However, the aerodigestive system is highly vulnerable to chemoradiotherapy toxicity and these treatments may lead to severe adverse events such as dysphagia. Dysphagia is one of the most important chemoradiotherapy-related side effects in head and neck cancer since it can lead to life-threatening complications such as aspiration pneumonia and malnutrition [3]. Dysphagia is defined as a swallowing disorder described as a “sensation of obstruction of the alimentary bolus” [4]. Considering its location, dysphagia can be classified as oropharyngeal dysphagia, which is often underdiagnosed, and its current screening is costly [4]. Oropharyngeal dysphagia involves disorders in the muscular mechanism of the pharynx and the upper esophageal sphincter or cricopharyngeal muscle. This disease is a common sequela in patients with an indication of non-surgical treatment of cancer, affecting up to 30–50% of these patients [5].

In Colombia, a low-/middle-income country, prior authors describe a prevalence of 64.28% of oropharyngeal dysphagia induced by head and neck cancer treatment (including surgical treatment) [6]. This study reported an average of 9 rehabilitation sessions per patient. Moreover, this study emphasized the high rates of financial barriers to medical care and rehabilitation in these populations, such as limited and delayed access to treatment, insurance administrative issues, transportation difficulties, low adherence to treatment, and low availability of appointments. Bright et al. described the inequalities in the distribution of otolaryngologists in most Latin American countries mainly due to a dearth of human resources [7]. Reports from the current literature suggest better swallowing outcomes with intensity-modulated radiotherapy (IMRT) compared to three-dimensional conformal radiotherapy (3DCRT) [3]. However, in Bogota, the capital city of Colombia, there are only four multidisciplinary teams registered in the healthcare database that perform IMRT which may only cover the needs of the local population [8]. There is no available information about the percentage of patients receiving conventional RT in comparison to IMRT in Colombia. In the Colombian clinical practice, conventional radiotherapy (3DCRT) is frequently used patients with head and neck cancer and this scenario leads to higher rates of adverse events related to chemo and radiotherapy. To date, there is limited scientific research in low-/middle-income countries describing the clinical challenges, related adverse events, and a review of the current literature. This information is essential to improve the outcomes in Latin American and low-income countries. This article aims to review the current scientific literature about dysphagia induced by chemo and radiotherapy and to compare this state of the art with the clinical practice challenges for otolaryngologists in Colombia.

## General Information on Radiotherapy and Chemotherapy in the Head and Neck Area

### Radiotherapy

In the early 1900s the pertinence of radiotherapy (RT) became controversial and lead to discussions due to its severe side effects [9]. However, the advances in radiation biology and the description of strategies to protect healthy tissues from these adverse effects led to a decrease in the incidence of adverse effects. In 1960 the development of linear accelerators marked the beginning of the modern era of RT [10]. Fractionated RT has been used since 1920 for head and neck tumors. Under the leadership of Gilbert Fletcher, surgery with moderate-dose RT became the standard treatment approach for locally advanced head and neck cancer. To date, adjuvant management with RT in head and neck cancer depends on the extent and stage of the tumor [10]. Advances in computer algorithms applied to imaging allowed an evolution of RT from two-dimensional plain x-ray images to three-dimensional x-ray-based images incorporating [11]. Conventional three-Dimensional Conformal Radiotherapy (3DCRT) targets the volume of adequate dose to the tumor with a minimum possible dose to normal tissue [11]. To date, further progress in conformal RT led to the development of Intensity Modulated Radiation Therapy (IMRT) that allows the optimization of the composite dose distribution with greater control within the target, improving the therapeutic radio [11, 12]. IMRT is planned by a radiation oncologist and a radiation physicist and is administered using a linear accelerator directly to a solid tumor. Unlike 3DCRT, the dose is precisely adjusted [13, 14]. Considering the high complexity and critical structures of the head and neck (i.e., salivary glands, optical system, inner ear, and brainstem), current studies suggest that IMRT has a significantly lower incidence of adverse effects and radiation toxicity compared to 3DCRT [12].

### Chemotherapy

A rigorous review of the indications for systemic treatment in patients with head and neck squamous cell carcinoma is beyond the scope of this manuscript. However, the authors of this review would like to highlight some of these indications and the most used agents. The treatment of locally advanced head and neck squamous cell carcinomas (HNSCCs) includes several therapeutic alternatives such as surgery. Surgery can be classified as open, transoral laser microsurgery, and robotic, the latter has been associated with less morbidity, higher local tumor control, and fewer complications related to extensive dissection [15]. In low- and middle-income countries, the use of robotics in surgery may help to reduce costs related to physician burnout, surgical site infections, and hospital stays [16]. However, due to the lack of potential socioeconomic factors such as high implementation costs and a lack of trained personnel may limit its accessibility and application [16]. An additional therapeutic option is a medical management which includes RT and systemic therapy (chemotherapy/CT). CT may have a role in the definitive treatment or can be used as an adjuvant treatment of RT.

Patients with HNSCCs with positive surgical margins or extracapsular invasion of resected lymph nodes have a worse prognosis and higher recurrence rates when treated exclusively with a surgical approach [15]. Thus, CT with three-weekly cisplatin plus RT is recommended as a first-line adjuvant treatment to improve locoregional control and overall survival [17]. However, cisplatin may enhance the toxicity of RT. In patients with cisplatin contraindication due to comorbidities, cetuximab is an effective therapeutic option together with RT and does not enhance RT toxicity (mucositis and radiodermatitis) [18, 19]. The main indications for cisplatin are (a) Concomitant with RT for the treatment of head and neck epidermoid carcinoma with local invasion; (b) As adjuvant treatment with postsurgical radiation for perilymph, perineural and extracapsular invasion; (c) CT induction with Docetaxel and 5-Fluoracil. Similarly, cetuximab can be used in (a) and (b) scenarios and can be additionally used for Head and Neck squamous cell metastatic carcinoma [17].

The most frequent histological type in head and neck neoplasms is squamous cell carcinomas [20]. Squamous cell carcinomas can be an aggressive tumor in advanced stages and may lead to cervical lymph node metastases. Therefore, combined therapeutic measures such as surgery with chemotherapy and radiotherapy (RT/CT) are essential. The estimated survival rates for each type of neoplasm, their head and neck stages, and the indications for RT are summarized in Table 1 [10]. Human papillomavirus (HPV) is frequently diagnosed in head and neck tumors, particularly in the oropharynx. The epidemiology of HPV around the world may have significant variations: the prevalence of HPV in the United States of America ranks from 40 to 80%, whereas its prevalence in European countries such as Spain can be 20% [2, 6]. The presence of HPV in patients with squamous cell carcinoma of the head and neck improves the prognosis but does not change the therapeutic management [18, 19]. HPV-positive tumors (P16-positive) have a better prognosis than HPV-negative tumors since tumor carcinogenesis in P16-negative patients is mainly environmental and related to tobacco and alcohol consumption [21]. Due to the molecular characteristics of HPV negative tumors (P53 mutations), these tumors may be more locally aggressive and may lead to lymph nodes and distant metastases [21]. The possibility of decreasing the intensity of systemic treatment and RT on HPV-positive tumors has been proposed to decrease the adverse effects without jeopardizing the tumor-free survival rate, locoregional control, and control of distant metastases [19].

**Table 1:** Treatment with radiotherapy and survival according to tumor histology

Type of neoplasm with its clinical stage	Treatment	5-year locoregional control rate	5-year survival rate
Nasopharynx carcinoma I II III-IV	RT: 66gy RT: 70gy (CT/RT): 70gy	95% 80% 90%	90% 84% 60%
Salivary gland carcinoma I-IV	Surgery + RTA^a^	I-II: 85% III-IV:55%	I-II: 82% III-IV:51%
Oropharynx I II III-IV	Surgery o RT 70gy Surgery o RT 70gy Surgery o CT/ RT	90% 85% 50%	70% 70% 30%
Larynx I II III IVa IVb	Surgery o RT: 66 Gy Surgery o RT: 70 Gy Surgery o CT/RT: 70gy Surgery/CTA/RTA^a^ o CT/RT	95% 80% 78% 80% 50%	80% 77% 30–60% 43% 30%
Carcinoma of unknown primary III III-IVa IVb	Without ECC: Node lymph dissection without RT Node lymph dissection / RT: 66 Gy CT/RT: 70 Gy/ Node lymph dissection	65% 90% 70%	62% 38–64% 32%
Skin carcinoma (lips, ear, nose)	Surgery, consider RTA^a^	95–99%	70%

^RT: Radiotherapy, CT: chemotherapy, RTA: Adjuvant radiotherapy, CTA: Adjuvant chemotherapy, ECC: Extracapsular extension, TORS: Transoral robotic surgery, MTL: Transoral Microsurgery with laser^

^a^ Adjuvant management required if: peri-lymph node invasion or perineural extension, surgical borders are positive for tumor, or extracapsular invasion

Another indication for definitive treatment with RT/CT in locally advanced head and neck squamous cell carcinoma based on “organ preservation”, which refers to the maintenance of the organ functionality [22]. Preserving functions such as swallowing, phonation, and respiration may depend on the location of the primary tumor. However, if there is tumor destruction of the organ to be “preserved”, surgery would be a better option. Among the “organ preservation” approaches, the induction CT followed by concurrent CR/RT (sequential CR/RT) and without induction. Induction RT is indicated in HNSCCs with lymph metastases and a high risk of distant metastases. Nevertheless, the benefits of induction RT remain unclear, as the studies are contradictory about overall survival and recurrences when compared to concurrent RT/CT [23, 24]. Moreover, the use of induction CT increases toxicity and decreases adherence to therapy, leading to a delay in treatment. Therefore, the use of sequential RT/CT should only be prescribed to patients with good general conditions and a good support network who present a high risk of locoregional recurrence and distant metastases [23]. Finally, CT is also used for palliative purposes in patients with tumor recurrence and distant metastases.

### Radiotherapy- and Chemotherapy-Induced Dysphagia in Head and Neck Cancer

**Fig. 1:**
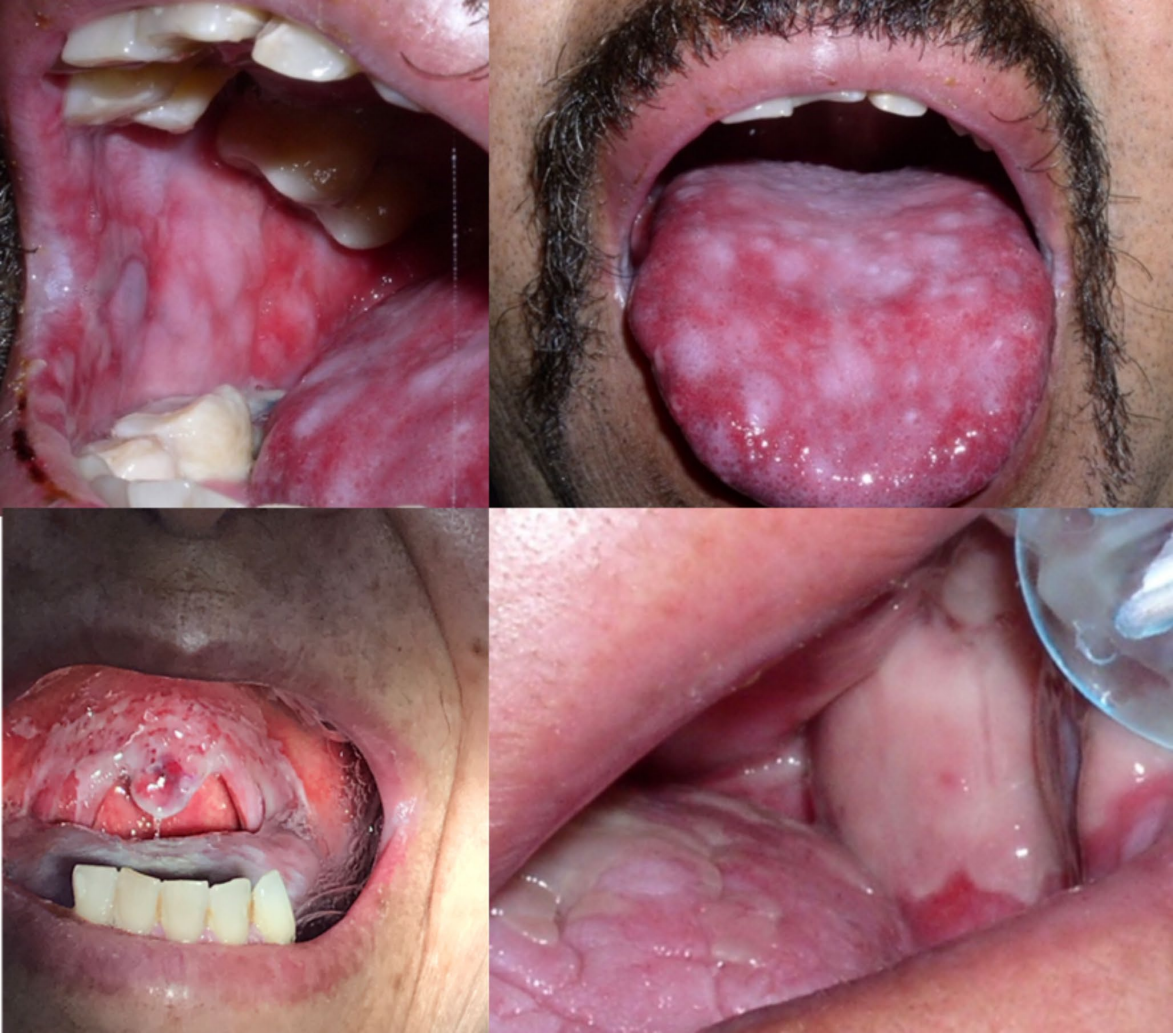
Grade III mucositis induced by cancer treatment. All patients provided written informed consent and agreed to the use of these pictures for educational and research purposes

**Fig. 2:**
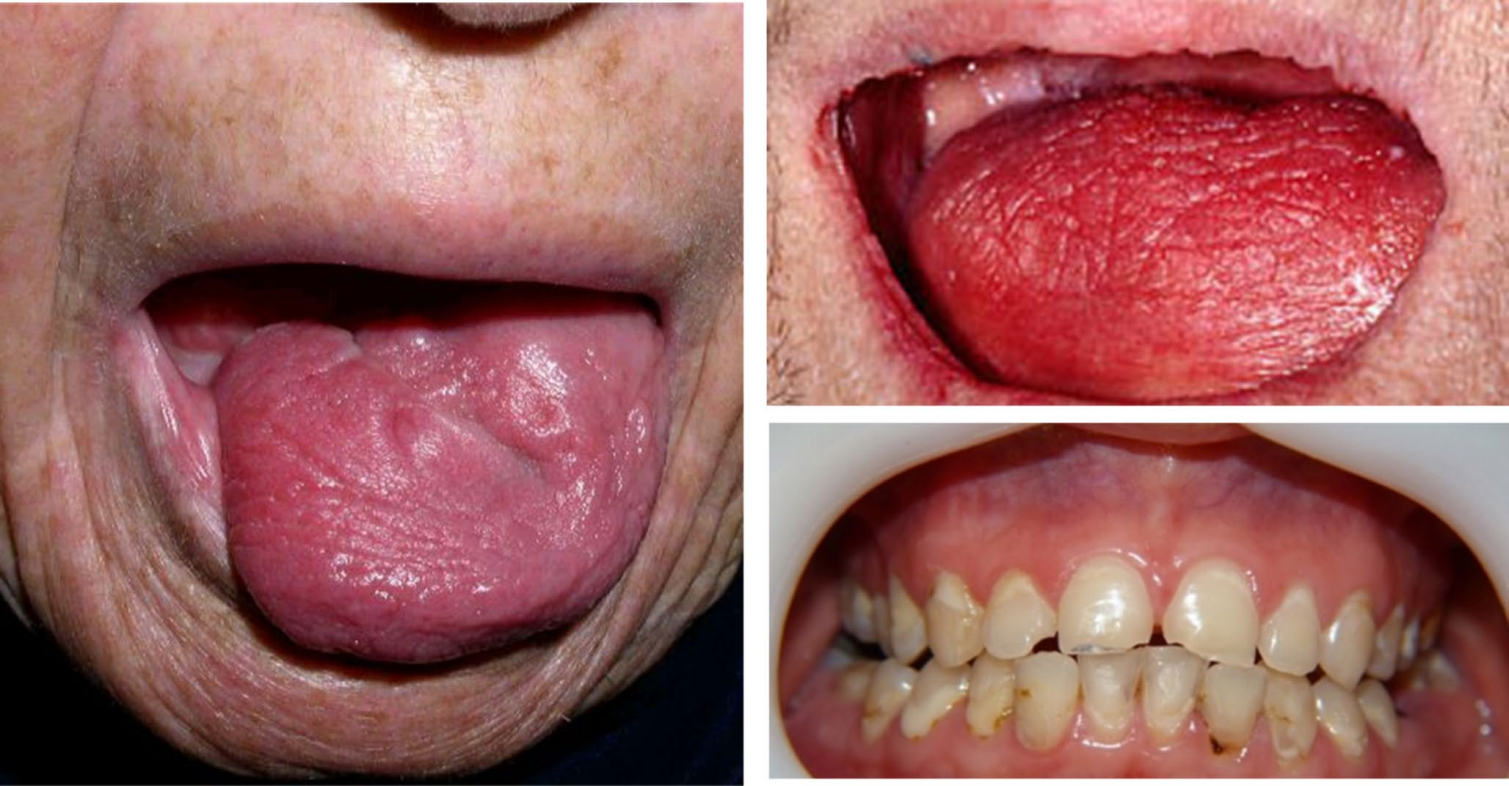
Xerostomia secondary to radiotherapy. All patients provided written informed consent and agreed to the use of these pictures for educational and research purposes

Oropharyngeal dysphagia is a common morbidity following head and neck RT/CT, with a prevalence of 30–80%. Dysphagia is most common in the first 3 months of treatment and has significant improvement after 6 to 12 months following completion of RT/CT in terms of weight gain, diet scores, gastrostomy tube-dependency and video swallow findings of aspiration or stenosis. [25]. However, up to 16% of patients may develop severe or progressive dysphagia several months after completion of management [25]. Moreover, dysphagia improvement can be assessed through objective tests such as instrumental assessment (video fluoroscopy and/or fiberoptic endoscopic evaluation of swallowing, FEES) [26]. Nevertheless, the access to these tests in low-/middle-income countries is limited due to budget limitations [27]. In Colombia, the follow-up of these patients is performed using questionnaires to assess dysphagia. Additional factors induced by RT/CT toxicity that may worsen dysphagia include xerostomia (most frequent), mucositis, tooth loss, nausea, fatigue, dysgeusia, and neck fibrosis [28]. Table 2 summarizes some of the effects of toxicity generated by RT/CT that may have a significant impact on swallowing. Figure 1 shows the clinical presentation of different patients with xerostomia induced by RT/CT, while Fig. 2 shows different patients with Grade III mucositis secondary to cancer treatment, these pictures were taken 1 month after RT/CT during follow-up at an otolaryngology referral center in Bogotá, Colombia.

**Table 2:** Factors that may worse CT/RT-induced dysphagia

Clinical findings	Affected organ	Time of appearance after CT/RT
Xerostomia	Salivary glands	2 weeks
Dysgeusia	Taste buds	4 to 6 weeks
Mucositis	Pharyngeal and larynx mucosa	2 weeks
Fatigue		7 days
Nausea		3 weeks

### Pathophysiology of RT/CT-Induced Dysphagia

**Fig. 3:**
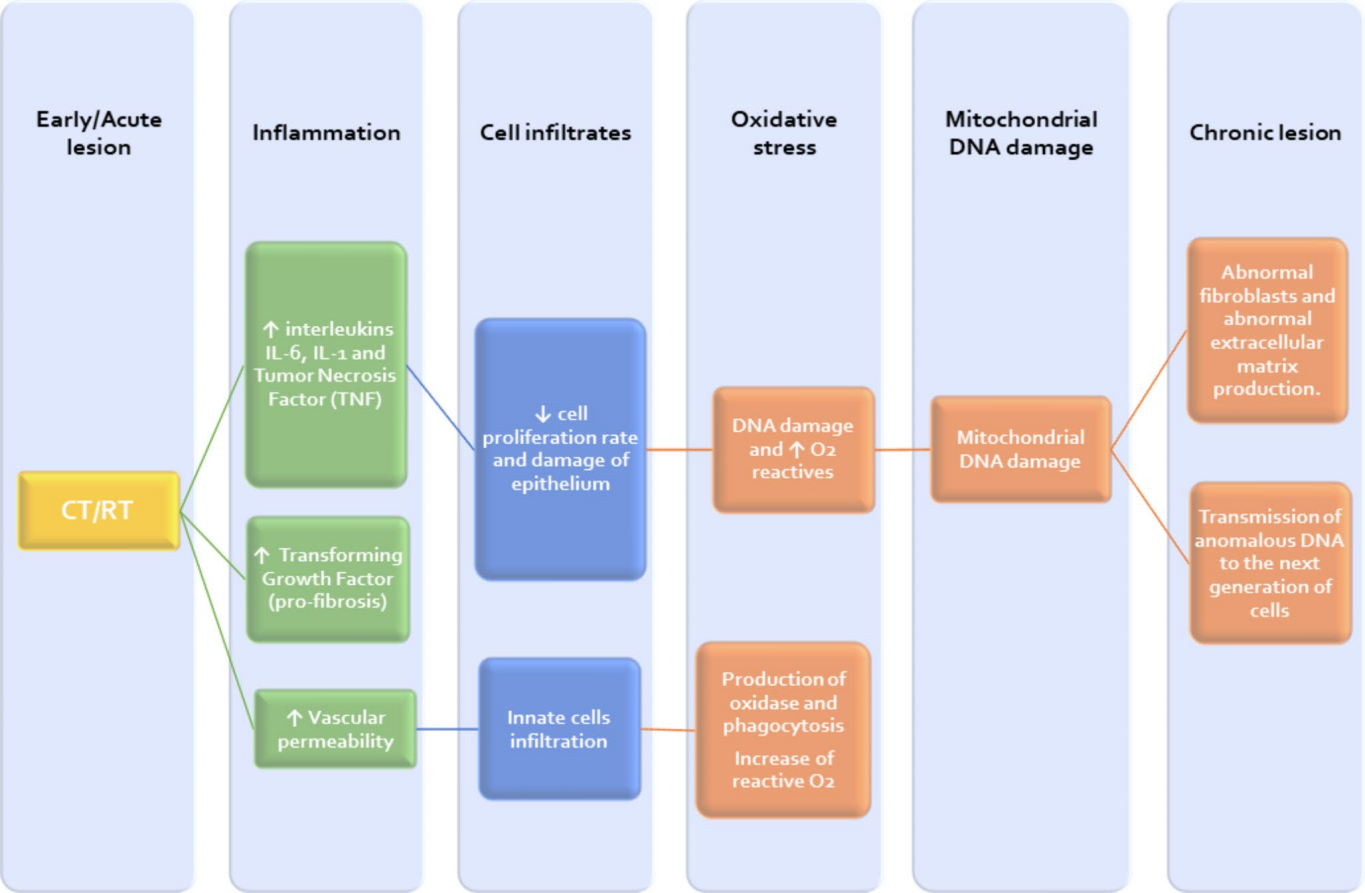
Pathophysiology of early and late chemoradiotherapy lesions

Swallowing is a complex process that involves the inhibition and activation of 25 different muscles. This process has 4 main stages that are briefly described in Table 3 [29]. RT and CT injuries depend on multiple factors such as the volume of radiation, the fraction of radiation dose, the total treatment time, and the total dose administered. The effects of radiation on healthy tissues can be classified as early and late effects (Fig. 3) [28]. The spectrum of acute effects may occur during the first three months of treatment, can be progressive, and usually resolve 3 to 4 weeks after the end of the treatment [9]. Head and neck radiotherapy schedules may take up to 7 weeks with a daily intensity of 5 days per week and dysphagia symptoms may begin in the first week. However, such symptoms may decrease due to the regenerative ability of healthy tissues [30]. Early effects are mainly attributed to oxidative stress, cell death, and inflammation [31]. These effects induce a storm of pro-inflammatory cytokines and chemokines that increase the vascular permeability, leading to edema [28]. Post –radiotherapy edema affects the larynx, pharynx, and the upper airway, decreasing the size of the swallowing channel, and leading to weakening of the muscles, which will compromise the bolus transport. Furthermore, the airway protective structures like arytenoid cartilage and epiglottis, may lose their function [28]. Early harmful effects are mainly attributable to the impact of radiotherapy on the mucosa, leading to mucositis, erythema, ulcers, impairments of taste buds, and decreased salivary secretion [31]. All these acute changes may be worsened by CT [32].

**Table 3:** Swallowing physiology

Swallowing phase	Description	
Oral preparation Voluntary phase Craneal nerves: V, VII and XII.	**Liquids**	**Solids**
	The tongue takes a dome position and holds the liquids. Contraction of the palatoglossal sphincter (uvula and base of tongue) to prevent passage of liquids into the oropharynx.	Solid foods are chewed and softened with saliva until they are converted into a bolus.
Oral phase Voluntary pase: Mainly regulated by the cerebral cortex	The base of the tongue descends and propels the liquid and bolus into the oropharynx. The soft palate rises and prevents the passage of the bolus into the nasopharynx.	
Faringeal phase Involuntary phase: Reflex arc: 1. Sensory fibers of the V, VII, IX and X pair 2. 2. Medulla oblongata: nucleus of the solitary tract and the nucleus ambiguus. 3. Efferent fibers of the V, VII, X and XII par.	The swallowing reflex starts when the bolus comes into contact with the anterior pillars (trigger zone). The food bolus passes into the pharynx through the vallecula. Elevation of the larynx, contraction of the pharyngeal constrictor muscles and displacement of the epiglottis posteriorly to cover the glottis and direct the bolus into the pyriform sinuses. Contraction of the supraglottic region and closure of the vocal cords, protecting it from food aspiration. Increase of pharyngeal pressure, generating an opening of the upper esophageal sphincter.	
Esofagic phase Involuntary phase	Peristalsis causes the bolus to enter the stomach through the opening of the lower esophageal sphincter.	

On the other hand, late or chronic lesions are those that persist or appear beyond 3 months after the end of radiotherapy [32]. Some acute lesions may persist and lead to chronic changes but there is no correlation between the severity of the acute lesion and the appearance of chronic lesions. Progression to chronic lesions is caused by delayed re-epithelialization that may trigger infection or tissue trauma (i.e., mucositis that develops into ulceration or necrosis). Late reactions produce connective tissue damage that causes abnormal cellular behavior leading to fibrosis formation [32, 33]. Early mucosal inflammation (xerostomia and mucositis) and radiation dose correlate with the occurrence of dysphagia 6 to 12 months post-treatment or even up to 2 years later [33]. Alterations in the muscles and nerves involved in swallowing are summarized in Table 4.

**Table 4:** Main findings in the swallowing muscles

Histologic changes (15)	Microvascular damage. Increased fibrosis and decreased elasticity leading to atrophy. Increased thickness of the extracellular matrix limiting muscle mobility.
Functional changes (21)	Decreased salivary gland function (Xerostomia). Decreased tongue base contraction and epiglottis retroflexion. Decreased pharyngeal mobility. Reduced synchrony between pharyngeal contractions, laryngeal closure and opening of the upper esophageal sphincter. Delayed or incomplete closure of the larynx during swallowing Delayed opening of the upper esophageal sphincter: chronic finding Decreased intrapharyngeal pressure, inhibiting with the movement the alimentary bolus Decreased pharyngeal sensation
Changes in the swallowing muscles and nerves	Injury of the type IIB (fast twitch) muscle fibers: These fibers use glycolytic metabolism for energy, have less mitochondrial formation, and are more prone to oxidative damage. Thus, the fiber are particularly susceptible to RT-induced damage. Acute inflammation increases the thickness of the pharyngeal constrictor muscle which limits its functionality (21). Destruction and disuse of the swallowing muscles during oncologic treatment (e.g. due to pain) contributes to the transformation from fibrosis to muscle atrophy (22). Irritation of the pterygoid muscles and temporomandibular joint leads to reduced oral aperture (23). Thinning and fibrosis of the mucosal layer may affect both afferent and efferent nerve terminals.

### Risk Factors Associated with RT/CT-Induced Dysphagia

Prior studies have described the risk factors for the development of RT/CT-induced dysphagia during therapy. The main factors are concomitant use of RT and CT [32]; advanced tumoral stage [22, 24]; the presence of dysphagia before starting treatment due to obstruction or tumor infiltration [25, 34, 35]; malnutrition and smoking; tumor localization (i.e. base of the tongue, larynx, nasopharynx); and a tumor located in the hypopharyngeal muscles which are more superficial, and therefore more susceptible to radiation damage. A correlation between the radiation dose and volume have been described encompassing the patient’s swallowing musculature with the development of dysphagia [36, 37]. Prior authors described an association between the radiation dosage applied and the elevated risk of subsequent aspiration and dependence on gastrostomy [37]. The risk of aspiration significantly increases when the larynx or inferior constrictor muscle is exposed to radiation doses exceeding 50 Gy [38]. Moreover, the likelihood of gastrostomy tube dependency increases when the laryngeal tissue is exposed to radiation doses surpassing 40 Gy, or when the targeted tissue volume receiving 60 Gy exceeds 24% [38]. Finally, it is essential to consider the type of radiation therapy as a significant risk factor [36, 37]. In comparison to conventional radiotherapy, IMRT can reduce radiation exposure to normal tissue while delivering precise radiation doses to tumor tissue, reducing the risk of swallowing dysfunction [37].

### Evaluation of Post-RT/CT Dysphagia

The initial assessment of the oncologic patient with dysphagia is similar to the assessment of dysphagia due to any cause [39]. A complete clinical history is required to evaluate the patient’s perception of the problem and to use validated clinical scales. It is always essential to perform an objective assessment of swallowing since subjective complaints may not correlate with the findings of an objective test, and up to 50% of patients may have a silent aspiration of food [40]. The information obtained with the tools described above will guide the identification of the location of dysphagia (i.e., oropharyngeal vs. esophageal), as well as to diagnose whether its etiology is structural (e.g., tumor compression) or functional.

Regarding the resource limitations of post-RT/CT dysphagia in Colombia, some hospitals and institutional boards have proposed protocols for follow-up of these patients [6]. However, some of the main challenges for these patients are related with administrative limitations (i.e. administrative procedures of the insurance companies that delay the diagnosis, follow-up appointments, and treatments), lacking specialists’ appointments, and transportation from rural areas to the main urban centers of the country. As an illustration, Maldonado et al. reported that despite 36 patients presented a clinical diagnosis of post-RT/CT oropharyngeal dysphagia, only eight patients achieved follow-up and access to rehabilitation services [6]. Moreover, in this study, the patients described that despite some insurance companies providing authorizations for up to ten sessions of follow-up and rehabilitation sessions, the administrative procedures must be restarted every two to three months, affecting timely care. In addition, the socioeconomic conditions of the patients led to low adherence to follow-up and treatments, as well as desertion of the therapeutic process [6]. A current alternative solution to these issues that some Quaternary Care hospitals are implementing in Colombia after the COVID-19 pandemic is clinical follow-up through telemedicine. However, this solution requires higher investment and are not easily available for most of the rural population. This scenario highlights the urgent need to develop guidelines with health jurisdictions aiming to provide clinical tools to primary care practitioners for disease surveillance, referral, and follow-up of higher risk patients.

### Clinical History and Physical Examination

Before surgical or medical treatment, patients with head and neck cancer may have different degrees of swallowing disorders and aspiration risk [41]. Overall, patients can have reduced tongue base retraction, reduced laryngeal elevation, delayed initiation of swallow, impaired laryngeal vestibule closure, and oral and pharyngeal residues [41]. Xinou et al. described that swallowing disorders prior treatment which may lead to further post-treatment deterioration or exacerbation of preexisting disorders [26]. Regarding the clinical history data, the otolaryngologist must inquire the patients about the presence of difficulties in chewing or swallowing. The presence of cough after ingestion of food as well as repeated pneumonia and oropharyngeal dysphagia must be considered as a main differential diagnosis. Usually, functional abnormalities have a gradual onset [42]. Patients may report difficulties swallowing all types of food and improvement of these symptoms with compensatory maneuvers. On the other hand, structural changes exhibit a more progressive onset, and the patient reports greater difficulty with solid foods. It is essential to use clinical scales that assess the subjective symptoms of the patient to monitor the symptoms such as Validity and Reliability of the Eating Assessment Tool (EAT-10) and the M.D. Anderson Dysphagia inventory (MDADI) [43]. In addition, all patients should have a nutritional assessment, a phono-audiology assessment, evaluation of voice quality and respiratory physiology, as well as a complete examination of cranial nerves V, VII, IX, X, and XII.

### Objective Examination

The most used objective examinations in clinical practice are the endoscopic evaluation of swallowing and videofluoroscopy [17]. Using these tools oropharyngeal dysphagia can be explained under three different models: (1) Infective or weak swallowing with incomplete clearance; (2) Swallowing through an incorrect pathway; (3) Delayed swallowing due to incoordination. Regardless of the model, these findings can help otolaryngologists to determine the presence of aspiration or food penetration, which is the most important risk factor for the development of aspiration pneumonia. Additionally, complementary tests such as electromyography of the cricopharyngeal muscle and esophageal manometry can be performed to assess esophageal motility [17, 44].

## Consequences of Rt-Induced Dysphagia

### Impact on Quality of Life

Treatment-induced morbidity in head and neck cancers has become increasingly important as patient survival has increased [40]. Dysphagia leads to increased time for feeding and changes in diet that can result in anorexia, weight loss, and fatigue. In addition, feeding is a social activity, and an eating disorder can lead to isolation [45]. Quality of life can be understood as the person’s perception of his or her state of health and involves social, psychological, and functional characteristics [46]. Evaluation of health-related quality of life is an essential aspect of clinical assessment and health research. Moreover, the measurement of quality of life is an important feature to guide therapeutic decision-making in clinical practice [25]. Questionnaires can be used to assess dysphagia in these patients, and some of them are validated in the Spanish Language such as Swallowing Quality of Life questionnaire (SWAL-QOL), the MD Anderson Dysphagia Inventory (MDADI), Dysphagia Handicap Index (DHI), and the Eating Assessment Tool (EAT-10) [43, 45]. Several studies suggest that quality of life can be compromised by dysphagia following the administration of CT and RT in the head and neck region [47, 48]. For instance, Campos et al. described the swallowing dysfunction and associated factors after treatment with RT and CT in patients treated for head and neck cancer using the SWAL-QOL [48]. This study described that the location of the tumor in the mouth was statistically associated with the lowest quality of life in the symptom’s domain (*p* < 0.05), and that the total dose of radiation received above 7000 Gy was associated with worse scores in the sleep domain (*p* = 0.008) [48]. Therefore, these factors should be considered as part of the clinical evaluation of these patients.

### Aspiration Pneumonia

Food aspiration can occur before, during, or after swallowing. Some triggers that may lead to food aspiration include premature dropping of food into the pharynx before vocal cord closure, inefficient glottic seal during swallowing, and an increase in food residues at the end of swallowing which let these remains fall into the airway [42]. However, a decrease in the sensitivity of the upper airway can cause silent aspiration. Prior studies state that up to 28% of patients who have received RT develop food aspiration in the first 3 months of treatment and the prevalence of aspiration decreases 6 months after the end of RT/CT [42, 49]. Moreover, food aspiration is the main risk factor for pneumonia and a total of 19% of non-cancer-related deaths are attributed to aspiration [5].

## Six Pearls to Reduce the Risk of Dysphagia Induced by Chemoradiotherapy

1. Perform a pre-treatment and/or on-treatment evaluation by a nutritionist and speech pathologist to identify patients who have swallowing or weight alterations that can be compensated with therapy before or during QT/RT [42].
2. Diet counseling and nutritional monitoring during CT and RT [42].
3. Adhere to maximum radiation doses to the swallowing organs as tumor size and location allow: average dose to the larynx should be less than 35 Gy; avoid irradiation to uninvolved pharyngeal constrictor muscles (dose less than 40 Gy) [49].
4. Poor nutritional status may affect the effectiveness of the treatment, so high-protein formulations and hypercaloric nutritional supplements are required to optimize nutritional status if needed. If oral nutrition becomes insufficient during treatment, a nasogastric tube or gastrostomy may be used [39].
5. There is no clear indication for the use of prophylactic gastrostomy before surgery. It should only be considered if the patient presents severe swallowing before the start of treatment [39].
6. Use of preventive swallowing exercises during therapy decreases the degree of muscle disuse atrophy and thus decreases the risk of dysphagia in the long term. Rehabilitation exercises directly target the masticatory, lingual, and oropharyngeal muscles through stimulation and exercise. In addition, these exercises can be complemented with postural measures, food viscosity adaptation, and oral hygiene [42].

## Rehabilitation of Rt-Induced Dysphagia

**Fig. 4:**
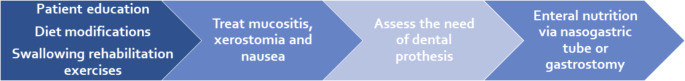
Process of rehabilitation of RT/CT-induced dysphagia

The rehabilitation of dysphagia requires a multidisciplinary team made up of otolaryngologists, oncologists, radiotherapists, nutritionists, speech therapists, psychologists, and social workers. This is to manage and support the patient in all biopsychosocial spheres and to identify the factors that may influence the improvement or worsen of dysphagia. The step-by-step rehabilitation is described in (Fig. 4) [50]. It is mandatory to provide enough education to the patients about their condition and teach them to identify and manage additional adverse effects derived from RT/CT toxicity that could worsen the dysphagia (e.g., mucositis, xerostomia, nausea) [42]. Moreover, an evaluation of the need for dental prostheses is particularly important [44]. If the patient reports xerostomia, some salivary substitutes, and gustatory stimulants such as sweet tastes and parasympathomimetic drugs (pilocarpine) can be useful. For the management of mucositis, it is essential to keep the oral cavity clean and free of food residues using saline solution and sodium bicarbonate, as well as avoiding irritating foods such as acidic and spicy foods [42, 49]. The use of mucosal lubricants that create a protective barrier such as oral ointments can be added. Furthermore, it is important to perform adequate pain management using topical analgesics such as lidocaine, antifungals, and antacids [42].

Finally, it is necessary to create a specific diet for each patient to evaluate which consistencies are easier to handle for the patient [39]. Avoiding foods that may trigger pain such as acidic and spicy foods, as well as mixed consistencies of foods and dry foods, are also essential steps since these foods could hinder the descent of the bolus. Concomitant to diet planning, swallowing rehabilitation is one of the most important steps to reduce the risk of aspiration [39].

## Challenges of Rt/Ct-Induced Dysphagia in Low-/Middle-Income Countries

To date, there is few scientific literature about dysphagia in low-/middle-income Latin American countries. In Colombia, Frias et al. reported that 1.17% of patients with dysphagia in a gastroenterology center in Bogotá had dysphagia secondary to radiation therapy [51]. In low- and middle-income countries, most patients do not have access to comprehensive swallowing rehabilitation for swallowing disorders [6]. Oncological follow-up and rehabilitation can be challenging for both the patients and the healthcare professionals, as conducting targeted therapies, and securing appointments for them is neither easy nor timely. The early initiation of an alternative nutrition route such as a gastrostomy is frequently chosen, and swallowing rehabilitation becomes a privilege for certain patients [6]. Furthermore, Bright et al. described the inequalities in the distribution of otolaryngologists in most low-/middle-income Latin American countries mainly due to a dearth of human resources [7]. Colombia, like most Latin American countries, faces several barriers in healthcare access that significantly impact the provision of quality care by otolaryngologists [7]. One of the primary challenges is the geographical distribution of health facilities, which tends to be concentrated in urban areas, leaving rural populations with limited access to healthcare [6, 52]. Thus, it can take months for a patient to be evaluated by an otolaryngology specialist, and an evaluation by a speech therapist with expertise in swallowing is also extremely limited. Furthermore, socioeconomic disparities affect access to healthcare, as individuals from low-income backgrounds are less likely to have health insurance or be able to afford medical services [6]. The lack of resources and infrastructure in some areas can also impact the quality of care provided, with inadequate diagnostic and therapeutic tools available to healthcare providers [52]. These barriers collectively lead to therapeutic challenges for otolaryngologists, as patients may not receive timely and appropriate treatment for their conditions, leading to potentially severe health complications. In terms of oncologic treatment, by the time patients are evaluated by a specialist, the patient is already at an advanced stage, which limits the therapeutic strategies and leads to significant surgical challenges.

In Colombia, prior authors describe a prevalence of 64.28% of oropharyngeal dysphagia induced by head and neck cancer treatment (including surgical treatment) and high rates of financial barriers to medical care and speech rehabilitation [6]. To date, in Bogota, the capital city of Colombia, there are only four multidisciplinary teams registered in the healthcare database that perform IMRT which may only cover the needs of the local population. Therefore, in the Colombian clinical practice, conventional 3DCRT is frequently used patients with head and neck cancer, which probably leads to higher rates of adverse events related to chemo and radiotherapy. Overall, this scenario may reflect the healthcare challenges in most low- to middle-income countries worldwide. However, there is limited scientific research in low-/middle-income countries describing the clinical challenges and related adverse events. Studies addressing these issues should be encouraged to improve the outcomes in low-income Latin American countries.
